# Effects of different altitudes on the structure and properties of potato starch

**DOI:** 10.3389/fpls.2023.1111843

**Published:** 2023-04-12

**Authors:** Tingyuan Hu, Hongkun Yang, Kaiqin Zhang, Cheema Nazir Hafsa, Xiaoting Fang, Haiyan Ma, Jiangxiu Liao, Shunlin Zheng

**Affiliations:** ^1^State Key Laboratory of Crop Gene Exploration and Utilization in Southwest China, College of Agronomy, Sichuan Agricultural University, Chengdu, China; ^2^Crop Ecophysiology and Cultivation Key Laboratory of Sichuan Province, Chengdu, China; ^3^Key Laboratory of Tuber Crop Genetics and Breeding, Ministry of Agriculture, Chengdu Joyson Agricultural Technology Co., Ltd, Xingdu, China

**Keywords:** altitude, potato starch, fine structure, chain length distribution, thermodynamic properties

## Abstract

The main element influencing the quality of potato starch is the environment. To investigate the effects of different altitude cultivation locations on the molecular structure and physicochemical properties of starch, two potato varieties, Jiusen No.1 B1 and Qingshu No.9 B2, were planted in three different altitude zones: A1 at low altitude (Chongzhou 450 m), A2 at middle altitude (Xichang 2800 m), and A3 at high altitude (Litang 3650 m). The results showed that the average volume, number, surface area diameter, average branched polymerization degree, crystallinity, and gelatinization temperature of two potato granules in high altitude areas were significantly lower than those in middle and low altitude areas were, and the gelatinization performance of potato starch was affected according to the correlation of starch structure characteristics. Potato starch with more short-branched chains and less long branched chains resulted in a lower gelatinization temperature in high altitude areas. The results showed that Jiusen No. 1 and Qingshu No. 9 were mainly affected by accumulated radiation and accumulated rainfall in Litang, a high altitude area, and by effective accumulated temperature in Xichang, a middle altitude area. This study quantified the influence of meteorological factors on the main starch quality of potato tubers. The results can be used as a theoretical basis for the scientific planting of high-quality potatoes.

## Introduction

The potato is a solanaceae plant, which is known as one of the most promising economic crops in the 21st century. Zhou (2008), is rich in starch, protein, vitamins, trace elements, and other ingredients with high nutritional value (Calliope et al., 2018; Li Hongxia et al., 2021). The United Nations Food and Agriculture Organization named wheat, corn, rice, and potato the four staple food raw materials in 2008 (Li X. et al., 2021). The potato originated in the high-altitude Andean mountains of South America and was then gradually introduced to the low-altitude plains. Because of the characteristics of climate and light in high-altitude areas, the characteristics of potato growth and development, potato formation, and so on are determined. With the introduction of potatoes into low-altitude areas, the quality of potatoes has also changed greatly. Because of this, altitude has always been an important factor in how potatoes taste and how their starch has made, so it is especially important to study how altitude affects potato starch. But it also affects the genetic make-up and physiological traits of the variety itself, differences in ecological conditions (such as temperature, light, rainfall, soil, etc.), and cultivation factors (planting density, fertilisation, pests and diseases, etc.) (Lombardo et al., 2013; Licciardello et al., 2018; Lombardo et al., 2020). The final quality of the tubers is influenced by a variety of growth and development stages, with ecological elements and agricultural cultivation factors having an impact on the potato's development at each stage of growth.

Medium starch is the main component of the potato tuber, which is between 66% and 80% by dry weight basis (Akhila et al., 2022). Moreover, potato starch has a unique quality and function that other starches can’t replace and is widely used in food processing, the chemical industry, medicine, textiles, papermaking, feed, and other fields. However, the structure and properties of potato starch are closely related. Potato starch consists of two main macromolecules: amylopectin (AP) and amylose (AM). AM is a major linear molecule with only a few branches, while AP is a very large and highly branched molecule (Schirmer et al., 2013). In the study of starch isolated from potato varieties planted in Canada, the results showed that the difference in chemical composition and molecular chain length of potato starch might lead to different functional characteristics of potato dry matter and single variety starch (Liu et al., 2007; Lu et al., 2011). Zhu and Hao (2018) investigated the molecular structure of Maori potato starch and discovered that Maori potato amylopectin had the longest units and internal chains and the fewest short chains. Further correlation analysis showed that the amount of short B chain (fb1, degree of polymerization (DP) 12–24) was related to the starting temperature (TO) and peak temperature (Tp) (Tappiban et al., 2020; Tappiban et al., 2020). It is reported that there is a negative correlation between the number of A chains (fa, DP 6–12) of colored potato starch and cold paste viscosity (CPV), gelatinization temperature (PT), Tp, and conclusion temperature (Tc). It was also found that the B2 chain (fb2, DP 24-36) was positively correlated with the amount of hot paste viscosity (HTV), CPV, PT, To, Tp, and Tc (Martinez et al., 2018; Tong et al., 2018; Vamadevan and Bertoft, 2018; Li et al., 2021). But no agreement has been reached because the relationship between the potato’s structure and function is very complicated and may be different for different types of potatoes and different ways they grow.

In recent years, more attention has been paid to the influence of environmental conditions on the composition, structure, and properties of starch (Kaur et al., 2007; Schirmer et al., 2013; Chung et al., 2014; Pelpolage et al., 2016). Currently, (Ahmed et al., 2018) have shown that the physicochemical properties of potato starch are affected not only by genotype but also by external factors such as climate, soil, and management measures. According to Šimková et al., 2013; and Zhang et al., 2022), the amylose content of wheat is also affected by the weather and soil conditions during grain development (Bains et al., 2017). At present Kaur et al. (2007); Trung et al. (2017) have investigated that no matter what the amylose content is, the decrease in grain and potato growth temperature will lead to an increase in starch particle size and a decrease in gelatinization and transition temperature. (Protserov et al., 2002) believed that this situation might be because of the increase in growth temperature that is parallel to the annealing mechanism in starch, which leads to changes in double helix length, the thickness of the crystalline layer, and the rigidity of the amorphous region. As far as we know, the research on the structure and properties of different potato starches mainly focuses on the analysis of the structure and properties of common potato starch (Chen et al., 2013). However, the influence of the environment on the fine structure of starch has not been reported. Therefore, it is necessary to obtain more accurate and reliable molecular structures of amylose and amylopectin molecules in different environments and further study the structure and properties of potato starch.

Therefore, in this study, two kinds of potatoes, Jiusen No. 1 and Qingshu No. 9, were planted at different altitudes to study the effect of the fine structure of potato starch quality, amylopectin, on the physical and chemical properties of starch. Moreover, as far as we know, there is little research on the fine structure analysis of potato starch amylopectin, by environmental factor. The purpose of this study was to: 1) study the effects of different altitudes on the fine structure of potato starch; and 2) study the effects of different altitudes on the physicochemical and thermodynamic properties of potato starch. The results of this study provide new insights and can be used to improve the structure, properties, and quality of starch. In addition, this research provides important information for growers so that they can better choose the suitable planting place for potato starch quality and ensure that the characteristics of starch are the best for consumers.

## Material and methods

### Test material and cultivation management

### Experimental design

#### Test methods

The experiment was carried out in experimental bases in Sichuan Province (Puti Village, Yangma Town, Chongzhou City; Wudaoqing Township, Puge County, Xichang City; Jiuxiang Town, Hanyuan County; and Jiawa Town, Litang County). A two-factor randomized block experiment was adopted. The first factor was altitude change: A1 low altitude area (450 m in Chongzhou), A2 high-altitude area (2800 m in Xichang), and A3 high-altitude area (3650 m in Litang). Factor B was variety: B1 (Jiusen 1) and B2 (Qingshu 9). Three repetitions, 48 combinations (communities), each with an area of 11.2m^2^. The experiment adopted single-ridge planting in the field, with 4 rows of ridges in each plot, 30 cm ridge height, 20 potatoes in each row, 20cm×70cm row spacing, and 10-15 cm planting depth. The rest were managed in the field according to conventional cultivation methods. Refer to **Table 1** for sowing times and cultivation management.

**Table 1:** Cultivation management.

Variety	Test point	Chongzhou	Xichang	Litang
Jiusen No.1	Sowing period	January 1st	March 1st	May 8th
	Harvesting period	May 15th	July 25th	October 20th
Qingshu No.9	Ploughing and tilling method	Ploughing and tilling method		
	fertiliser	base fertilizer :40 kg of compound fertiliser (N/P/K=17/17/17) and 1000 kg of organic farmyard manure per mu; after the seedlings have emerged: 5 kg of nitrogen fertiliser (urea) per mu.		

#### Meteorological data

**Figure 1:**
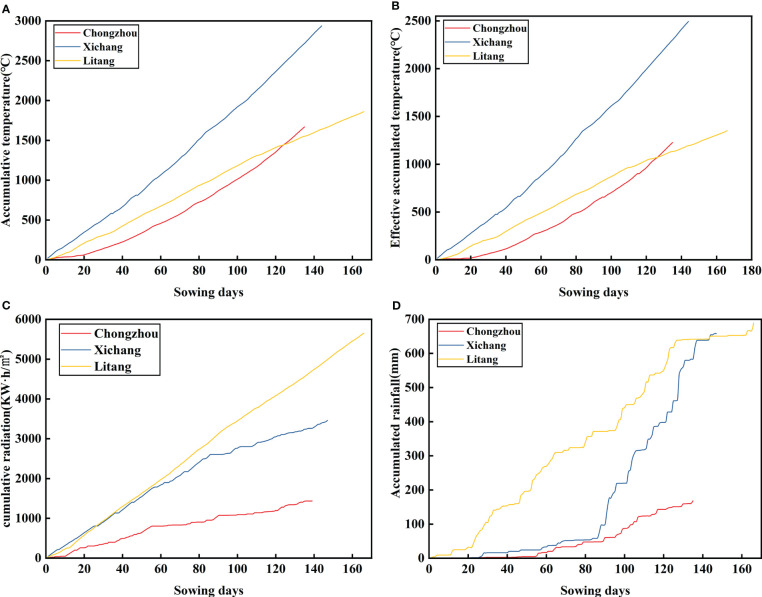
Cumulative temperature, cumulative effective accumulated temperature, cumulative radiation and cumulative rainfall in potato growth period at different altitudes. **(A–D)** respectively represents accumulative temperature, effective accumulated temperature, cumulative radiation and accumulative rainfall in different places.

As can be seen from **Figure 1**, the average temperature of Chongzhou was 13.23°C during the potato growth period in 2021; the accumulated temperature was 1667.4°C, the effective accumulated temperature was 1227.40°C, and the rainfall was mainly concentrated from March to April, with the accumulated rainfall of 127.70 mm and less sunshine at 398.60 h; the accumulated radiation was 1434.96 KWh/m2. During the potato growth period in 2021, the average temperature in Xichang was 21.60°C, the accumulated temperature was 2936.7°C, and the rainfall was mainly from June to July, with an accumulated rainfall of 269.98 mm and 961.80 h of sunshine; the accumulated radiation was 3462.84 kW h/m2, and the effective accumulated temperature was 2552.15°C. The average temperature in Litang during the potato growth period in 2021 was 10.85°C. The accumulated temperature was 1860.2°C, the accumulated rainfall, mainly from May to September, was 391.96 mm, and there were 2161.98 h of sunshine. The cumulative radiation was 5651.28 KWh/m2, and the sunshine was sufficient. The effective accumulated temperature was 1350.40°C. To sum up, during the potato growth period in 2021, the average temperature was Xichang > Chongzhou > Litang; cumulative temperature: Xichang > Litang > Chongzhou; precipitation: Litang > Xichang > Chongzhou; sunshine: Litang > Xichang > Chongzhou; cumulative radiation: Litang > Xichang > Chongzhou; and effective accumulated temperature: Xichang > Litang > Chongzhou. In the potato growth period of 2021, the cumulative precipitation, sunshine intensity, and cumulative radiation increased with the increase in altitude, and the cumulative effective accumulated temperature and cumulative temperature increased first and then decreased.

### Test methods

#### Sample pretreatment

Wash the appropriate amount of fresh potatoes and cut them into pieces, mash them with water in a tissue masher, sieve them to remove cellulose and other substances, and leave the starch sediment in water. To obtain a starch sample, wash them with distilled water 3–4 times, stand still for 6-7 hours, pour off the supernatant, remove the brown powder layer, and dry them in an air-drying oven at 40°C for 48 hours to obtain a starch sample. The sample was screened with a 100-mesh sieve.

#### Determination of starch composition

The content of starch in potato tubers was determined by anthracene copper chromogenic method (Zhang et al., 2019). The amylose content in tuber starch was determined by dual-wavelength calorimetry. Amylopectin content was obtained by subtracting amylose content from total starch content (Zeng et al., 2012; Wang et al., 2017).

### Analysis of starch granule morphology and particle size

Take a 2~3 g mature potato tuber, break it off with tweezers to obtain a cross section, cut it with a blade to obtain another flat cross section, and fix it on the sample with conductive adhesive. After gold spraying, the sample was directly observed by a scanning electron microscope (S4800II, Hitachi). A small amount of purified starch was picked up with tweezers and dispersed on the conductive adhesive. After gold spraying, it was directly observed by a scanning electron microscope. In order to compare the starch particle size distribution, the LS13320 laser diffraction particle size analyzer of Beckman Coulter Company analyzed starch particles. Put 50 mg of starch into a centrifuge tube, add 5 mL of distilled water for suspension, swirl and mix for 1 hour at 4°C, oscillate once every 10 minutes, then transfer to the dispersion box of a laser diffraction particle size analyzer, and measure its distribution.

### Analysis of starch-chain length distribution

For an examination of starch chain length distribution, Isoamylase (E-ISAMY, Megazyme, Ireland) was used to remove the branches of starch, and then the relative molecular weight distribution of starch was determined by high temperature gel exclusion chromatography (GPC, PL-GPC 220, USA). High performance liquid chromatography (HPAEC, Thermo ICS-5000, USA) was also used to look at the distribution of chain lengths in amylopectin (Zhang et al., 2017).

### Crystal structure analysis of starch

The relative crystallinity of starch was analyzed by a polycrystalline X-ray diffractometer (D8-ADVANCE, Germany). A Fourier transform infrared spectrometer (Varian 7000 FTIR, USA) was used to analyze the short-range ordered structure of starch. The crystal lamellar strength and thickness of starch were analyzed by a small-angle X-ray scatterometer (Li et al., 2016).

### Thermodynamic determination of starch

Measure the thermal characteristics of DSC6. Weigh 4 mg (dry basis) of starch or rice flour samples in an aluminum crucible (PE0219-0062), add deionized water at a mass ratio of 1:2, cover and seal, place at room temperature and balance overnight, and then measure. The empty crucible was used as the reference. The scanning temperature range was 20-140°C, and the scanning rate was 10°C/min. The temperature was 30 -120°C, and the thermal characteristic parameters and enthalpy (H) values were calculated. Two parallel samples were taken from each sample, and the results were averaged (Singh et al., 2003).

### Data analysis

The data were processed using statistical analysis software such as Microsoft Excel 2010 and SPSS 27, and a variance analysis was performed.

## Results

### Effect of different altitude on starch composition of potato

As shown in the data in **Table 2**, the content (%) of amylose and amylopectin and the ratio of amylose/amylopectin in potato tubers reached extremely significant levels among different varieties, different altitudes and the interaction between varieties and altitudes. At different altitudes, the starch content of dry sample of Jiusen No.1 and Qingshu No.9 was about 80%~86%, the amylose content of dry sample of Jiusen No.1 potato starch was about 26% ~ 31%, the amylopectin content was about 69% ~ 74%, and the amylose content of dry sample of Qingshu No.9 potato starch was about 25% ~ 31%, and the amylopectin content was about 69% ~ 75%. The starch content of both increased with the elevation. The amylose content of Jiusen No.1 was higher than that of Qingshu No.9. As the altitude increases, the straight-chain starch of Qingshu NO.9 shows a trend of first decreasing and then increasing.

**Table 2:** Variance analysis of the content (%) and proportion of potato starch, amylose and amylopectin of potato varieties at different experimental site.

Altitude	Variety	Starch	Amylose	Amylopectin	Amylose/amylopectin
Chongzhou Xichang	Jiusen No. 1	81.56c	29.44c	70.56c	0.42c
	Qingshu No. 9	80.85c	26.65d	73.35b	0.36d
	Jiusen No. 1	84.31b	30.97a	69.03e	0.45a
Litang	Qingshu No. 9	81.34c	25.37e	74.63a	0.34e
	Jiusen No. 1	86.22a	25.85e	74.15a	0.35e
	Qingshu No. 9	86.57a	30.12b	69.88d	0.43b
F-value	altitude treatment	14.82**	130.08**	129.84**	118.50**
	variety treatment	112.79**	235.13**	235.10**	220.30**
	altitude×variety treatment	11.50**	41.66**	41.62**	36.49**

Different letters in the data in the same column represent the significance reaching 0.05; starch content is the percentage of dry weight; ** represents the significance level of 1%; the following are the same.

### Morphological analysis of potato starch granules

**Figure 2:**
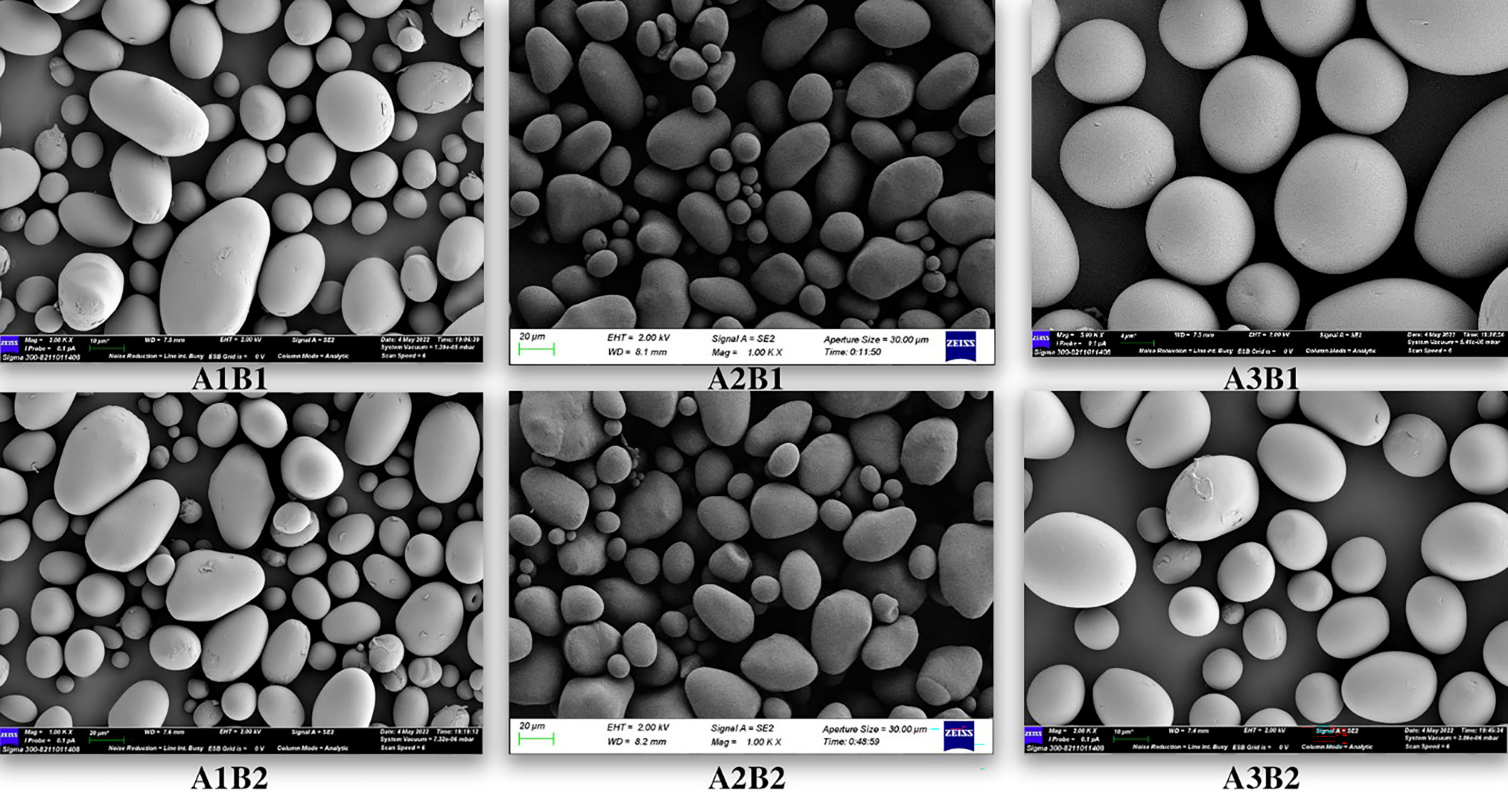
Electron microscope scanning images of potato starch granules at different altitudes. A1, A2 and A3 represent Chongzhou, Xichang and Litang respectively, B1 and B2 represent Jiusen No. 1 and Qingshu No. 9.

**Figure 2** showed an electron microscope scanning picture of potato starch granules with magnification. It was observed that potato starch granules were mostly oval and round and the starch granules of Jiusen No.1 and Qingshu No.9 were roughly the same in shape. At low altitude, some starch granules of Jiusen No.1 showed irregular shapes, but at high altitude, the shapes were more uniform. At low altitude, the starch granule surface of Qingshu 9 was very rough, with many irregular bulges, while at high altitude, the surface was smoother. In general, potato starch granules extracted from Gaohai were more regular in shape and smoother in surface.

### Particle size analysis of potato starch

**Figure 3:**
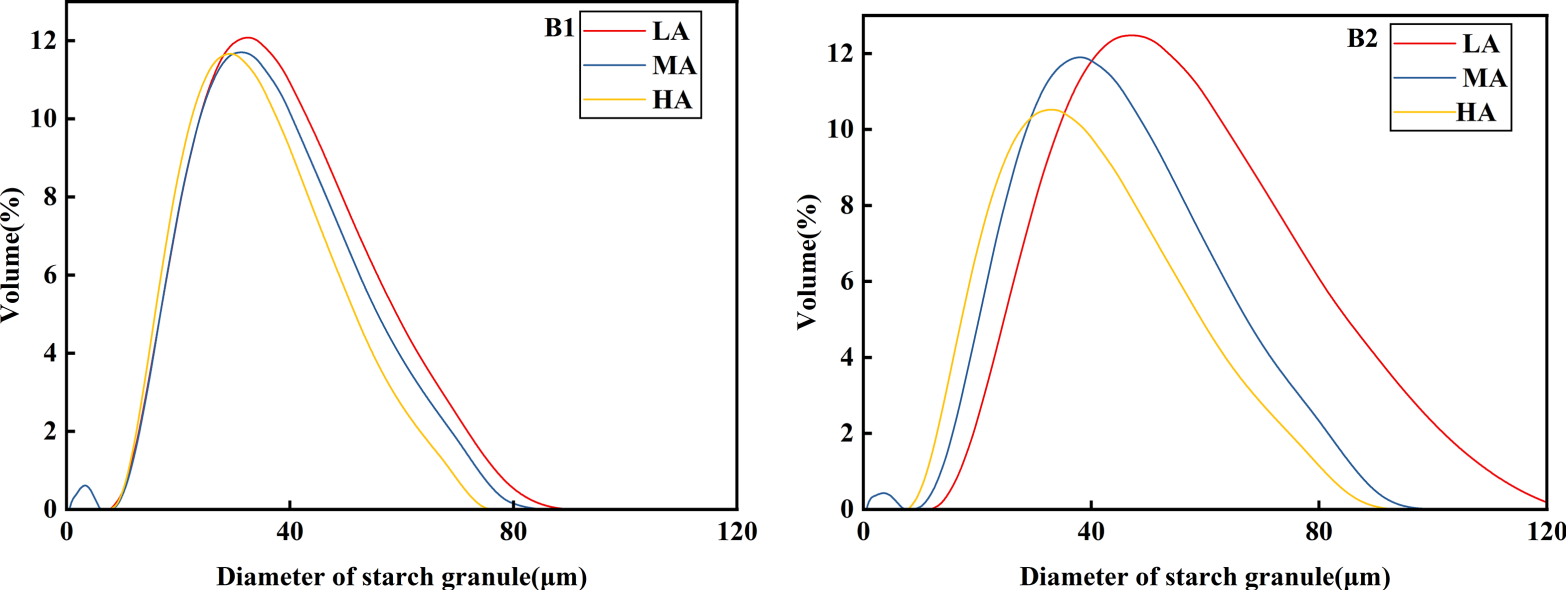
Volume distribution of potato starch granules at different altitudes. LA (low altitude), MA (middle altitude) and HA (high altitude) stand for Chongzhou, Xichang and Litang respectively; B1 and B2 stand for Jiusen No. 1 and Qingshu No. 9 respectively.

**Figure 4:**
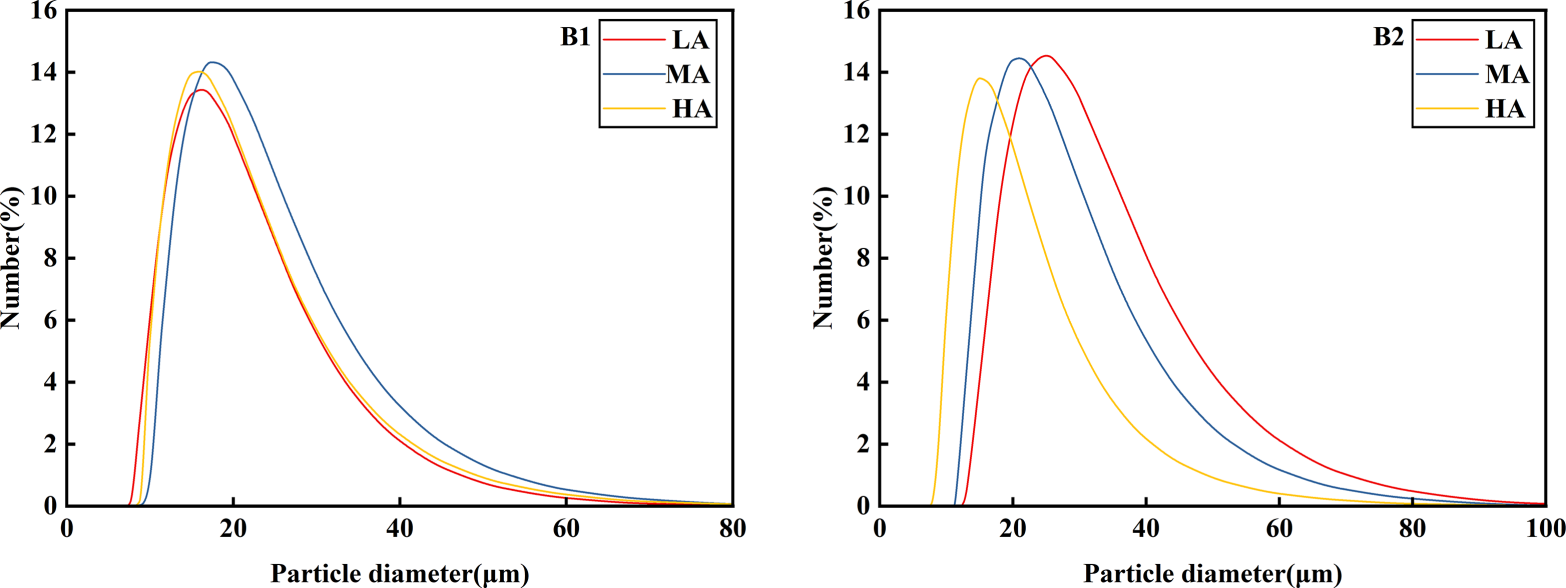
Number distribution of potato starch granules at different altitudes. LA (low altitude), MA (middle altitude) and HA (high altitude) stand for Chongzhou, Xichang and Litang respectively; B1 and B2 stand for Jiusen No. 1 and Qingshu No. 9 respectively.

**Figure 5:**
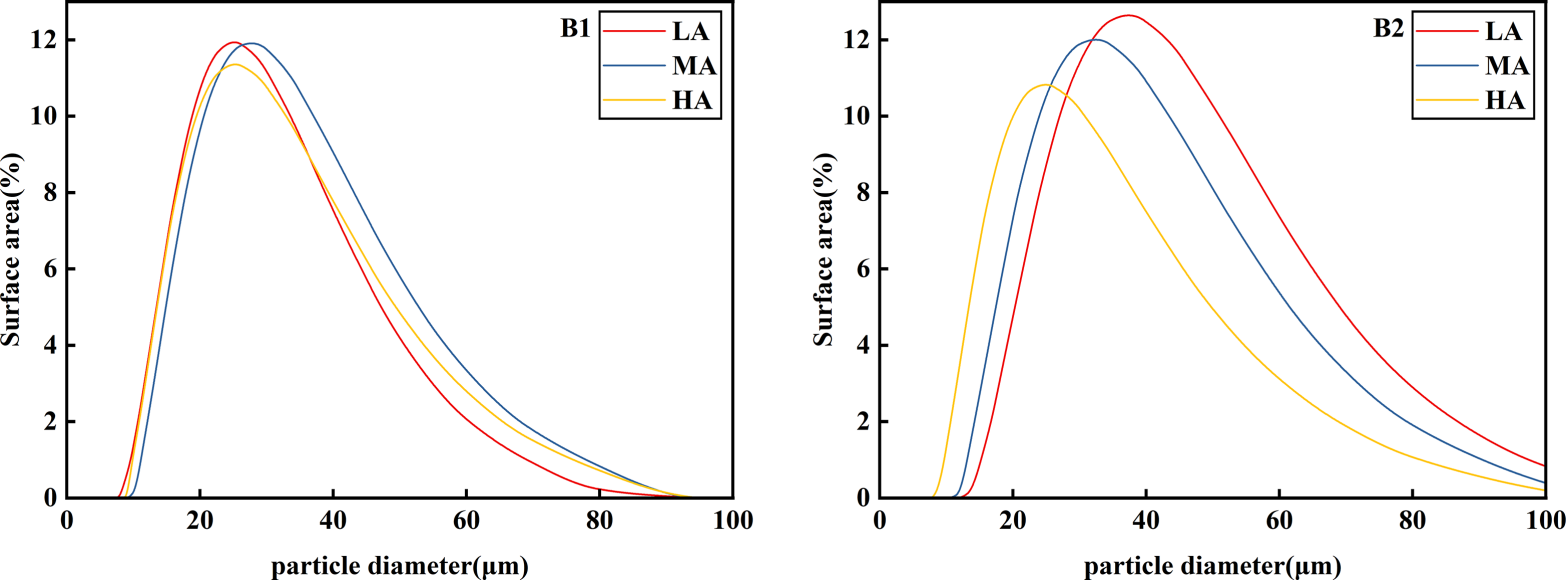
Surface area distribution of potato starch granules at different altitudes. LA (low altitude), MA (middle altitude) and HA (high altitude) stand for Chongzhou, Xichang and Litang respectively; B1 and B2 stand for Jiusen No. 1 and Qingshu No. 9 respectively.

According to **Figure 3** and **Table 3**, it can be seen that the potato granule volume was roughly unimodal, and there was a big difference in starch granule volume among different potato varieties. Qingshu No.9 was significantly larger than Jiusen No.1 was. With the increase of altitude, the starch granule volume changes of the two varieties were basically consistent, while Jiusen No.1 and Qingshu No.9 both decreased with the increase of altitude. According to **Figure 4**, at the same altitude, the number of starch granules of Jiusen No.1 was more than Qingshu No.9 in quantity distribution, and the difference of potato starch granules in low altitude was large, but the difference of potato starch granules in high altitude was small, which indicates that potato starch granules in high altitude were evenly distributed. According to **Figure 5**, in terms of surface area distribution, at the same altitude, the surface area of starch granules of Qingshu No.9 was larger than that of Jiusen No.1 with the increase of altitude, the surface area of starch granules of two varieties decreased significantly. Overall, the starch granules of the two potato varieties decreased with the elevation, and the average volume and surface area of the starch granules of Jiusen at all elevations were smaller than those of Junqingshu No.9.

**Table 3:** Diameter distribution of potato starch granules at different altitudes.

Altitude	Variety	Volume weighed mean diameter	Number weighed mean diameter	Surface area weighed mean diameter
Chongzhou	Jiusen No. 1	33.66d	19.37e	28.07b
	Qingshu No. 9	48.75a	29.84a	41.14a
Xichang	Jiusen No. 1	30.90e	21.99c	17.17d
	Qingshu No. 9	37.27b	25.77b	18.56c
Litang	Jiusen No. 1	30.50f	19.84d	15.66f
	Qingshu No. 9	34.09c	19.32e	16.98e
	altitude treatment	982.64**	78.00**	371.33**
F-value	variety treatment	2315.05**	165.86**	132.82**
	altitude×varietytreatment	398.98**	80.97**	44.58**

Different letters in the data in the same column represent the significance reaching 0.05; starch content is the percentage of dry weight; ** represents the significance level of 1%.

### Comparison of fine structure of potato starch at different altitudes

**Figure 6:**
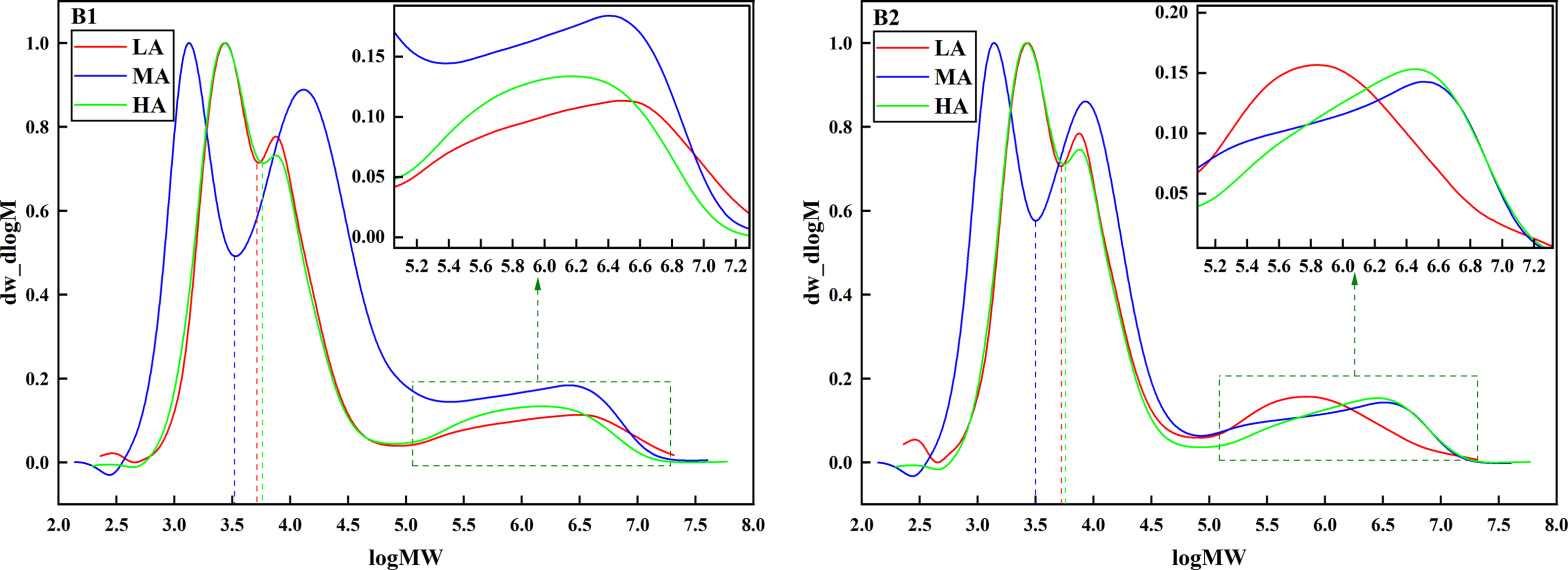
Relative molecular weight distribution of potato starch at different altitudes. LA (low altitude), MA (middle altitude) and HA (high altitude) stand for Chongzhou, Xichang and Litang respectively; B1 and B2 stand for Jiusen No. 1 and Qingshu No. 9 respectively.

In order to clarify the influence of starch fine structure on potato quality, the relative molecular weight distribution of these two potato starches was analyzed by high temperature gel permeation exclusion chromatography (GPC). As shown in **Figure 6**, the starches of the two samples were divided into three peaks. Based on the previous studies, it could be known that the peak 1 and the peak 2 were the short chain (AP1) and the long chain (AP2) of amylopectin respectively (Zhang et al., 2017). Among them, the tendency of AP1 components of Jiusen No.1 and Qingshu No.9 starch was the same, with the increase of altitude, AP1 first increases and then decreases. Studies has shown that AP1/AP2 reflects the branching degree of amylopectin, and the larger the ratio, the higher the branching degree of starch. By calculating the branching degree of two samples, it was found that the ratio of AP1/AP2 of Jiusen No.1 to starch (2.89 0.03) was significantly higher than that of Qingshu No.9 (3.09 0.02) at low altitude A1. The ratio of starch AP1/AP2 of Jiusen No.1 was lower than that of Qingshu No.9 at A2 and A3 altitudes. The third peak showed the amylose component (AM), from which it could be seen that the amylose component of Qingshu 9 was significantly more than that of Jiusen 1, especially the short chain component of amylose was more obvious.

### Effect of chain length distribution of potato starch amylopectin at different altitudes

**Figure 7:**
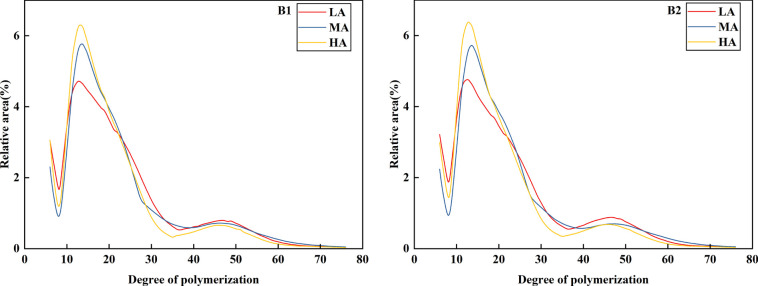
Distribution of amylopectin chain length of potato starch at different altitudes. LA (low altitude), MA (middle altitude) and HA (high altitude) stand for Chongzhou, Xichang and Litang respectively; B1 and B2 stand for Jiusen No. 1 and Qingshu No. 9 respectively.

In order to further clarify the chain length distribution characteristics of amylopectin, information was obtained by means of high performance liquid anion chromatograph (HPAEC). According to previous studies, the chain length distribution of amylopectin could be divided into four types: one short chain (A, DP6-12) and three long chains (B1, DP13-24;; B2, DP25-36; B3, DP > 36) **Figure 7** showed the chain length distribution diagram. The chain length distribution diagram of Jiusen No.1 and Qingshu No.9 was similar, with the highest peak at DP12-13 and double peaks. As shown in **Table 4**, at the altitudes A1, A2 and A3, the chain length ratio of Jiusen No.1 and Qingshu No.9 varieties A, B1 and A/B increased with the increase of altitude, while the chain length ratio of B2 and B3 decreased with the increase of altitude, showing an opposite trend. However, the average polymerization degree was highest in Xichang, which increases at first and then decreases with the increase of altitude. The starch chain length ratio of Jiusen No.1 and Qingshu No.9 was mainly concentrated in chain A and B1, with the ratio of 18.21%~24.28% and 44.68%~53.49% respectively. However, the proportion of B2 and B3 chains was small, and the short chain of Qingshu 9 was significantly higher than that of Jiusen 1 at different altitudes.

**Table 4:** Characteristics of chain length distribution, average chain length and polymerization degree of amylopectin in different treatments.

Altitude	Variety	A(DP≤12)	B1(DP 13-24)	B2(DP 25-36)	B3(DP≥37)	A/B	Average degree of polymerization
Chongzhou	Jiusen No. 1	21.77d	46.29e	16.95a	14.99d	0.278d	22.58d
	Qingshu No. 9	22.96b	44.68f	16.18b	16.18a	0.298b	22.72c
	**Mean**	**22.37C**	**45.49C**	**16.57A**	**15.59A**	**0.288B**	**22.65B**
Xichang	Jiusen No. 1	18.21f	52.25b	14.27d	15.27c	0.223e	22.78b
	Qingshu No. 9	18.23e	51.42d	14.88c	15.47b	0.223e	22.98a
	**Mean**	**18.22B**	**51.84B**	**14.58B**	**15.37B**	**0.223C**	**22.88A**
Litang	Jiusen No. 1	22.45c	53.49a	12.32e	11.74f	0.289c	20.79e
	Jiusen No. 1	24.28a	51.88c	11.78f	12.07e	0.321a	20.66f
	**Mean**	**23.37A**	**52.69A**	**12.05C**	**11.91C**	**0.305A**	**20.73C**
F-Value	altitude treatment	3572.28**	4546.56**	412.83**	424.10**	592.21**	1029.42**
	variety treatment	369.54**	1240.77**	451.71**	243.48**	102.01**	114.92**
	altitude×varietytreatment	101.08**	2287.67**	1298.07**	791.08**	265.81**	220.70**

Different letters in the data in the same column represent the significance reaching 0.05; starch content is the percentage of dry weight; ** represents the significance level of 1%. Mean represents the average value.

### Effect of different altitudes on crystal structure of potato starch

**Figure 8:**
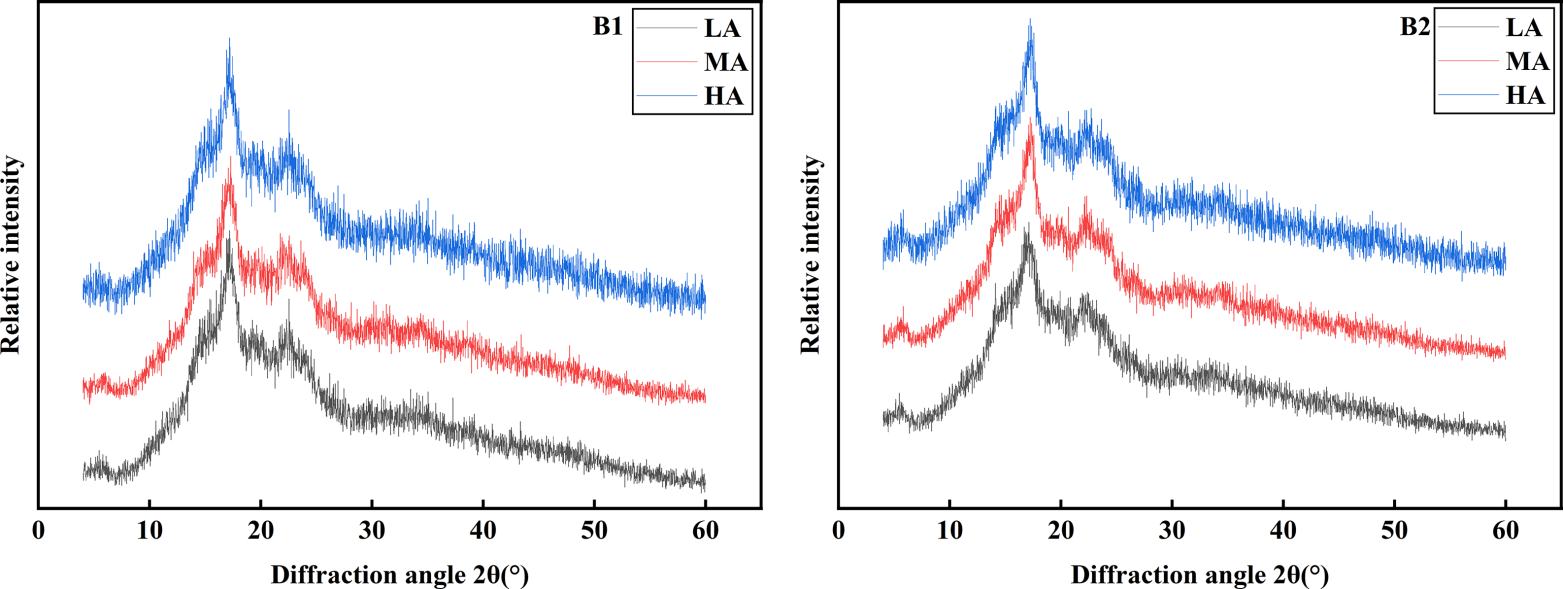
XRD patterns of potato starch at different altitudes. LA (low altitude), MA (middle altitude) and HA (high altitude) stand for Chongzhou, Xichang and Litang respectively; B1 and B2 stand for Jiusen No. 1 and Qingshu No. 9 respectively.

Potato granules are mainly semi-crystalline complexes with double helix crystal structure formed by amylopectin molecules and amorphous structure formed by some amylopectin and amylose, so the molecular structure of starch has an important influence on the crystal type and crystallinity of starch (Gan et al., 2009). Firstly, the crystal diffraction of two starch samples were analyzed by X- ray diffraction (XRD). **Figure 8** showed the X-ray diffraction patterns of two potato starches at different altitudes. From **Figure 8**, it can be observed that there was no obvious difference in the peak types of the two potato starches, with strong diffraction peaks around 5.5 ~ 5.6, 15.0, 19.7, 22.2 and 24.0 at 2θ angles. Therefore, it was be judged that this potato starch might be a B-type crystalline structure. This was the same as the research results of other varieties of potato starch reported in the literature (Chen et al., 2013). However, after further comparison and analysis of the long-range ordered structure (relative crystallinity) of the two starches, it was found that there were significant differences between the relative crystallinity of the two potato starches at different altitudes, and the relative crystallinity of Qingshu No.9 (21.42%, 48.30%, 24.84%) was significantly higher than that of Jiusen No.1 (15.05%, 39.75%) With the increase of altitude, the relative crystallinity of different potato varieties first increased and then decreased, and the relative crystallinity ranges from 15.05% to 39.75% and from 21.42% to 48.3% respectively. This result was in good agreement with the difference of fine structure of starch. Because the starch crystal is mainly composed of double helix structure formed by medium and long chains of amylopectin, it can be inferred that the relatively large number of long chains of amylopectin in Qingshu No.9 starch was the main reason for its high crystallinity. In addition, this also explains the high enthalpy value of Qingshu No.9 starch, because the higher the crystallinity of starch, the more heat it needs in the gelatinization process.

**Figure 9:**
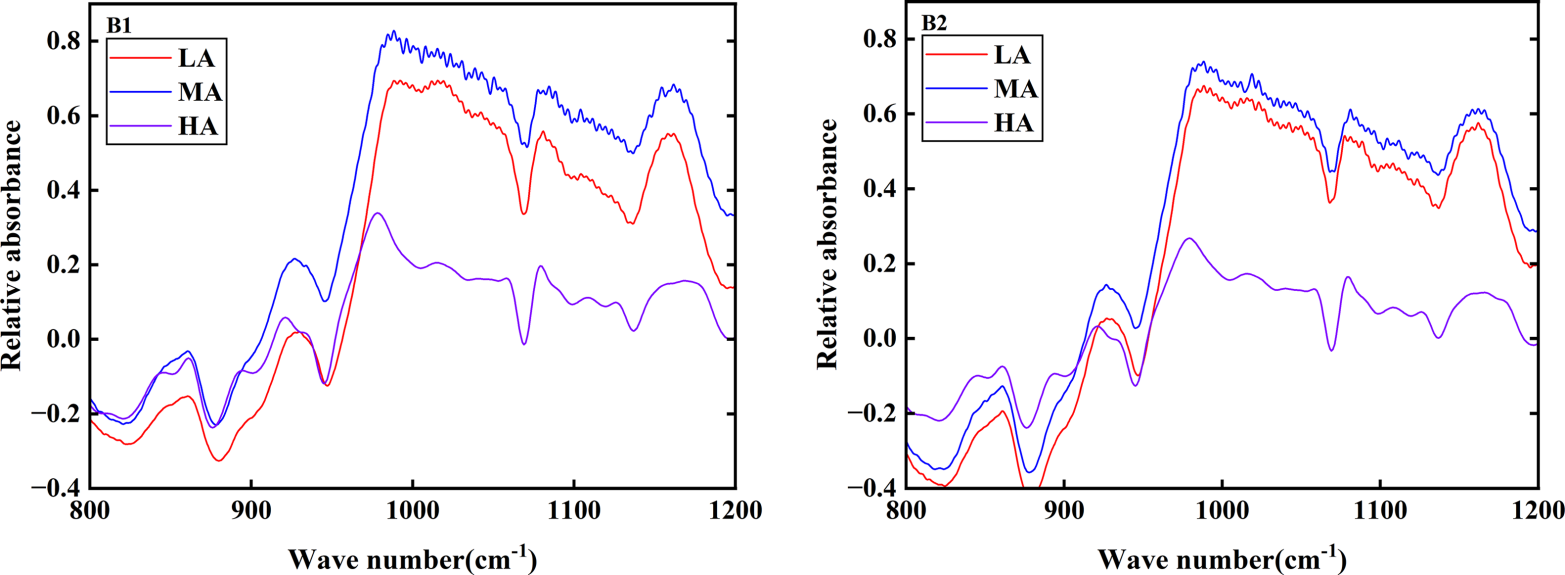
FTIR spectra of potato starch at different altitudes LA (low altitude), MA (middle altitude) and HA (high altitude) stand for Chongzhou, Xichang and Litang respectively; B1 and B2 stand for Jiusen No. 1 and Qingshu No. 9 respectively.

Fourier transform infrared spectroscopy can accurately analyze the short-range ordered structure of starch crystal structure. As shown in **Figure 9** and **Table 5**, the infrared absorption curves of Jiusen No.1 and Qingshu No.9 starch were consistent. Usually, the infrared absorption peaks of 1045/cm and 1022/cm in the infrared spectrum were the structural characteristics of starch crystallization region and amorphous region, and the ratio of them (1045/1022) can reflect the short-range order degree of starch molecules (Jay-lin, 2009). Through the calculation of characteristic values, it was found that the short-range order degree of Jiusen No.1 and Qingshu No.9 starch was significantly different at different altitudes, and the difference trend was consistent with the crystallinity, with the short-range order degree ranging from 0.81 to 0.94. At low altitude (Chongzhou), the short-range order degree of Jiusen No.1 starch was lower than that of Qingshu No.9 starch, and that of Jiusen No.1 starch was higher than that of Qingshu No.9 starch at other altitudes.

**Table 5:** Crystallinity and IR values of starch of two varieties at different altitudes.

Altitude	Variety	Crystallinity(%)	1045/1022cm^-1^	1022/995cm^-1^
Chongzhou	Jiusen No. 1	15.05f	0.87d	0.98a
	Qingshu No. 9	21.42d	0.89c	0.96b
Xichang	Jiusen No. 1	39.75b	0.94a	0.86d
	Qingshu No. 9	48.30a	0.91b	0.84e
Litang	Jiusen No. 1	19.32e	0.84e	0.95b
	Qingshu No. 9	24.84c	0.81f	0.91c
F-value	altitude Treatment	209.06**	144.90**	113.74**
	variety Treatment	37.60**	34.63**	260.26**
	altitude×variety Treatment	65.95**	123.65**	47.78**

Different letters in the data in the same column represent the significance reaching 0.05; starch content is the percentage of dry weight; ** represents the significance level of 1%.

### Effect of different altitude on thermodynamic properties of potato starch

According To the comparative analysis of **Table 6**, the thermodynamic properties of starch were determined as TO (initial temperature), Tp (peak temperature), Tc (conclusion temperature) and ΔH (enthalpy of crystal melting). All the results were shown in Table. These parameters reflect the proportion of crystals related to molecular order. Amylose content was considered as the main factor to control the thermal properties of starch, but it is not the only factor. Other factors affecting thermal properties include molecular structure of amylopectin/amylose and amylose lipid complex (Liu et al., 2006).

**Table 6:** Thermodynamic properties of potato starch at different altitudes.

Altitude	Variety	T0	Tp	Tc	ΔH (J/g)
Chongzhou	Jiusen No. 1	61.24c	65.29c	70.53c	13.78d
	Qingshu No. 9	60.76d	63.83d	68.35d	14.70c
	**Mean**	**61.00B**	**64.56B**	**69.44B**	**14.24B**
Xichang	Jiusen No. 1	62.60b	66.90b	74.00b	16.01a
	Qingshu No. 9	63.70a	67.70a	74.60a	15.26b
	**Mean**	**63.15A**	**67.30A**	**74.30A**	**15.64A**
Litang	Jiusen No. 1	58.17f	61.20f	66.91f	13.60f
	Qingshu No. 9	58.59e	61.76e	67.29e	13.66e
	**Mean**	**58.38C**	**61.48C**	**67.10C**	**13.63C**
	altitude treatment	6163.19**	9155.97**	1456.81**	11408.19**
F-value	variety treatment	97.28**	906.01**	129.67**	47.47**
	altitude×variety treatment	169.67**	416.18**	645.02**	188.23**

Different letters in the data in the same column represent the significance reaching 0.05; starch content is the percentage of dry weight; ** represents the significance level of 1%. Mean represents the average value.

**Figure 10:**
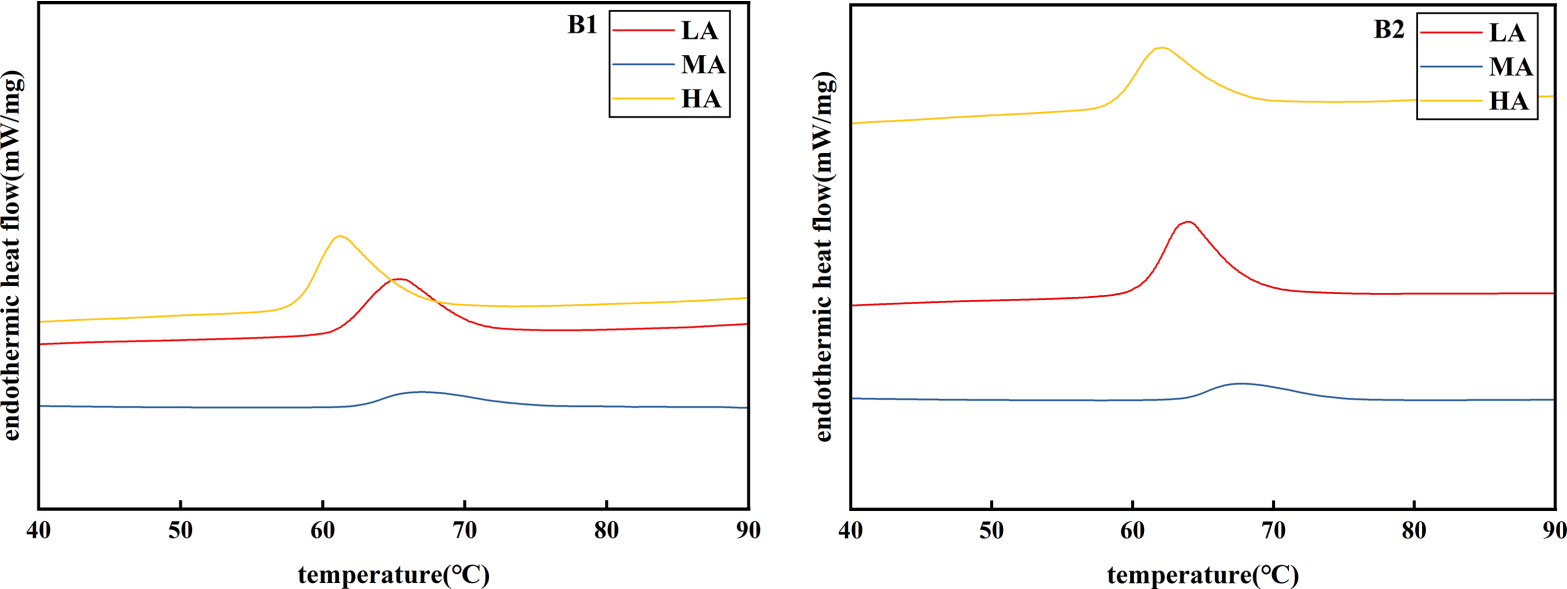
DSC endothermic curves of potato starch at different altitudes. Principal component analysis of starch indexes at different altitudes. LA (low altitude), MA (middle altitude) and HA (high altitude stand for Chongzhou, Xichang and Litang respectively; B1 and B2 stand for Jiusen No. 1 and Qingshu No. 9 respectively.

At different altitudes of Chongzhou, Xichang and Litang, the initial temperature, peak temperature, final gelatinization temperature and enthalpy change of Jiusen No.1 and Qingshu No.9 potato varieties all showed the same trend of first increasing and then decreasing. At all altitudes, the initial temperature, peak temperature, final gelatinization temperature and enthalpy change of Qingshu No.9 starch were slightly higher than those of Jiusen No.1 variety, and the changes were 58.17~63.70°C, 61.20~67.70°C and 67.29 ~74.60°C, respectively. In addition, because Qingshu No.9 starch has high enthalpy, it needs to absorbed more heat. **Figure 10** (DSC endothermic curve) of the thermal gelatinization characteristics of two potato starch varieties showed that in Xichang, the temperature span of Jiusen No.1 and Qingshu No.9 for gelatinization is longer, that is to say, the time required for the thermal gelatinization process is also longer.

### Principal component analysis of each component at different altitudes

**Figure 11:**
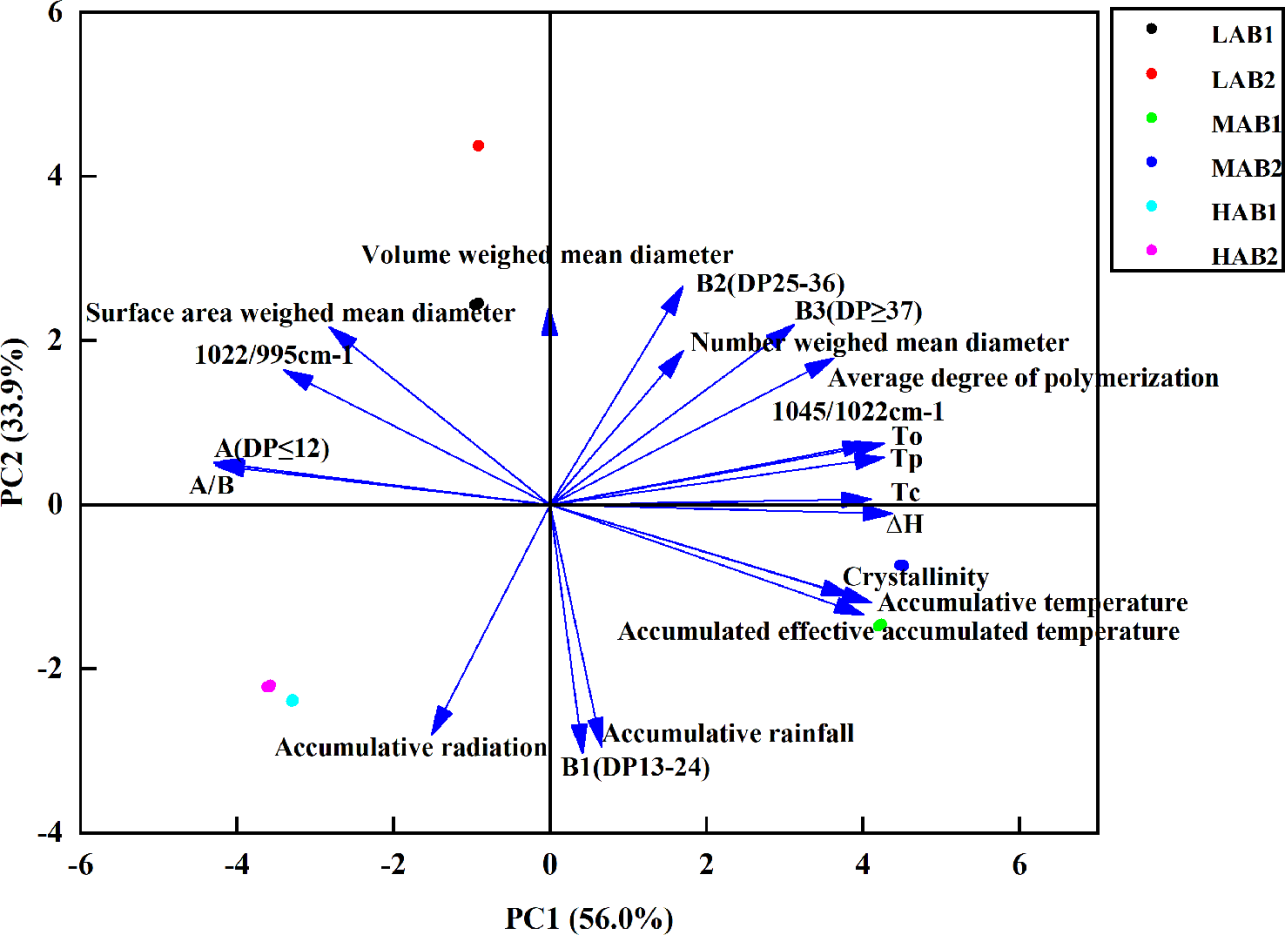
Principal Component Analysis of Potato Indicators at Different Altitudes. LA (low altitude), MA (middle altitude) and HA (high altitude stand for Chongzhou, Xichang and Litang respectively; B1 and B2 stand for Jiusen No. 1 and Qingshu No. 9 respectively.

Further dimension reduction analysis of potato starch indexes showed in (**Figure 11**) that the contribution rates of principal component 1(PC1) and principal component 2(PC2) were 55.0% and 33.9% respectively. The total variance contribution rate of them was 88. 9%, which means that PCA1 and PCA2 jointly explain 88.9% of the differences in the physicochemical properties and structure of potato starch at different altitudes. It could be seen from the figure that the spatial distribution of different altitudes was obviously different, indicating that the differences of starch indexes in altitude areas were significant due to meteorological factors, and there were also significant differences among varieties. Accumulated temperature, accumulated effective accumulated temperature were positively correlated with starch thermodynamic properties (To, Tp, Tc, H), polymerization degree, crystallinity, weighted average diameter, medium and long chain B2 (DP25-36), B3(DP≥37), 1022/995cm^-1^. Accumulated radiation and accumulated precipitation have significant positive correlation with short chain A. Jiusen No.1 and Qingshu No.9 were mainly affected by accumulated radiation and accumulated rainfall in Litang at high altitude, and by accumulated temperature and accumulated effective accumulated temperature in Xichang at middle and high altitude.

## Discussion

### Effects of different altitudes on potato starch morphology and particle size distribution

Starch is the main component of potatoes, and the starch content in different varieties of potatoes was significantly different. The starch content in fresh potatoes was 9%-23%, and that in dry potatoes was 66%~80%. It was divided into amylose and amylopectin. From the morphology of starch grains, Jiusen No.1 and Qingshu No.9 in this study have no obvious difference in starch shapes, both of which were round or oval. However, with the increase in altitude, the surface of starch was smoother and the number of small granular starches increases. Generally, starch granules were divided into ≤2μm (small starch granules), 2~5μm (medium starch granules) and ≥5μm (large starch granules) according to their particle size. In the particle size distribution of potato starch granules, different varieties of potato starch granules account for the largest proportion (Chung et al., 2014). However, this study believed that planting potatoes at low altitude and at high altitude was beneficial to increase the proportion of starch granules and reduce the average weighted body area number and surface area diameter of starch granules. The results of this study were consistent with those of its predecessors, and so is this study. The influence of temperature during grain filling on the particle size distribution of potato starch by predecessors was reported by (Liu et al., 2017). It also showed that the increase in the proportion of starch granules in rice grains was not conducive to the improvement of the cooking and eating quality of rice. Therefore, potatoes planted in high-altitude areas taste better.

### Effects of different altitudes on the chain length distribution of potato starch amylopectin

In general, high temperatures tend to raise the proportion of amylopectin with a long chain A chain and decrease the proportion with a short chain B chain, whereas low temperatures have the reverse effect. (Tao et al., 2020) reported how the environment affects the distribution of rice’s starch chain length; it was thought that the proportion of long-chain amylopectin was positively connected with nighttime temperatures while the proportion of short-chain amylopectin was negatively correlated. (Chung et al., 2019) reported that amylopectin with a shorter or less long chain lowers the gelatinization temperature (To and Tp). Additionally, (Du, 2019) demonstrated that the amount of amylopectin’s chain length has an impact on how easily starch gelatinizes. The gelatinization temperature of rice starch increases with the number of long amylopectin chains present and becomes more challenging to gelatinize. The retrogradation degree and retrogradation enthalpy rise concurrently, the rice hardens after cooling, and this is not advantageous for the enhancement of rice flavor quality. According to the findings, potatoes grown at low and medium altitudes considerably raised the proportion of long chain amylopectin and decreased the fraction of short chain amylopectin. The amount of long chain amylopectin significantly dropped while the amount of short chain amylopectin increased when potatoes were cultivated at a high altitude.

### Effects of different altitudes on the crystal structure of potato starch

The crystalline structure of starch from different sources was different. According to the different X-ray diffraction patterns, the crystalline structure of starch could be divided into three types, namely, type A, type B, and type C. The difference was that the rotation angle range of the strong diffraction peak was different (Zhong et al., 2017). The results of this study showed that potato starch has a B-type crystalline structure at different altitudes, which was consistent with previous studies. Some studies have shown that the order of the surface structure of starch could be judged by the ratio of crystallinity of 1045/1022 cm^-1^ and 1022/995 cm^-1^ (Zhou et al., 2020). Compared with low altitude, the crystallinity and the ratio of 1045/1022 cm^-1^ in the high altitude area were lower. Previous studies had shown that the high crystallinity of starch improved the stability of starch, lead to an increase in gelatinization temperature, and reduced the eating quality of rice (Lu et al., 2011; Zhang et al., 2011). Therefore, potatoes planted in high altitude areas had better tasting quality.

### Effects of different altitudes on thermodynamic properties of potato starch

The thermodynamic properties of starch were an important index to reflect the quality of starch, which was not only affected by the content of amylose and amylopectin but also by the chain length distribution of amylose and amylopectin (Chung et al., 2011). On the influence of ambient temperature on starch thermodynamic (DSC) characteristics, (Liu et al., 2017) considered that high temperature stress increases the gelatinization temperature of starch, and the main reason was that high temperature increases the proportion of large-sized starch and the size of starch granules. (Chun et al., 2015) considered that high temperatures during grain filling reduced the short chain ratio of amylose and amylopectin and increased the medium chain ratio of amylopectin, which leads to the increase in the gelatinization temperature of starch. (Neoh et al., 2020) reported that cold-treated wheat grains significantly affect the starch structure, and the gelatinization temperature of starch becomes lower, which affects the function of flour and the quality of final products. (He et al., 2010; Zhu, 2018) showed in their correlation analysis that the distribution ratio of small starch granules were negatively correlated with gelatinization temperature, while the proportion of large starch granules and the diameter of starch surface area were positively correlated with gelatinization temperature. The relative crystallinity and peak strength of starch were positively correlated with gelatinization temperature, enthalpy, and retrogradation enthalpy. This study showed that compared with potatoes in high altitude areas, potatoes plucked from low and middle altitude areas have more large starch granules and larger starch granule diameter. The higher the gelatinization temperature, the higher the relative crystallinity and peak strength of starch, and the higher the gelatinization heat. It was consistent with previous studies. Therefore, the amount of starch and starch granules in high altitude areas was higher, the gelatinization temperature was lower, and the amount of heat needed for gelatinization was less. This makes high altitude areas a better place to plant potatoes to make them taste better.

## Conclusion

Growing potatoes at various elevations had a substantial impact on the starch structure and qualities of potatoes, according to a research of the starch structure and characteristics of two potato kinds. The starch structure and characteristics of Jusen 1 and Green Potato 9 revealed notable variations between varieties, altitudes, and interactions between poster and variety. While Xichang is mostly influenced by cumulative temperature and cumulative effective cumulative temperature at intermediate and high altitudes, Litang is primarily influenced by cumulative temperature and cumulative radiation at high altitudes. Higher altitude Litang saw a rise in the proportion of small starch grains and a decrease in the average starch grain size compared to lower altitudes, while Chongzhou saw the opposite trend. Rice's starch crystal types are unaffected by altitude, but at higher altitudes, Litang increases the proportion of short chains of branched starch, decreases relative crystallinity and peak starch intensity, and lowers the temperature and enthalpy of starch pasting, which results in more delicately flavored potato varieties. Because of the higher cumulative radiation and cumulative rainfall at medium and high altitudes compared to low and medium altitudes, potatoes grow better and are more suitable for agriculture there. The findings of this study offer a critical point of reference for the production of potatoes at various elevations in Sichuan Province, which will aid in making the best use of ecological resources and enhance potato farming quality.

## Data availability statement

The original contributions presented in the study are included in the article/supplementary material. Further inquiries can be directed to the corresponding author.

## Author contributions

All authors contributed to the article and approved the submitted version.
